# Characterization of Blow-Spun Polyurethane Scaffolds–Influence of Fiber Alignment and Fiber Diameter on Pericyte Growth

**DOI:** 10.1021/acsbiomaterials.4c00051

**Published:** 2024-06-10

**Authors:** Iwona Łopianiak, Aleksandra Kawecka, Mehtap Civelek, Michał Wojasiński, Iwona Cicha, Tomasz Ciach, Beata A. Butruk-Raszeja

**Affiliations:** † Laboratory of Biomedical Engineering, Faculty of Chemical and Process Engineering, Warsaw University of Technology, Waryńskiego 1, Warsaw 00-645, Poland; ‡ Doctoral School of Warsaw University of Technology, Plac Politechniki 1, Warsaw 00-661, Poland; § Section of Experimental Oncology und Nanomedicine (SEON), Else Kröner-Fresenius-Stiftung-Professorship, ENT-Department, Universitätsklinikum, GluckstraBe 10a, Erlangen 91054, Germany

**Keywords:** aligned fibers, mechanical properties, solution blow spinning, polyurethane, pericytes

## Abstract

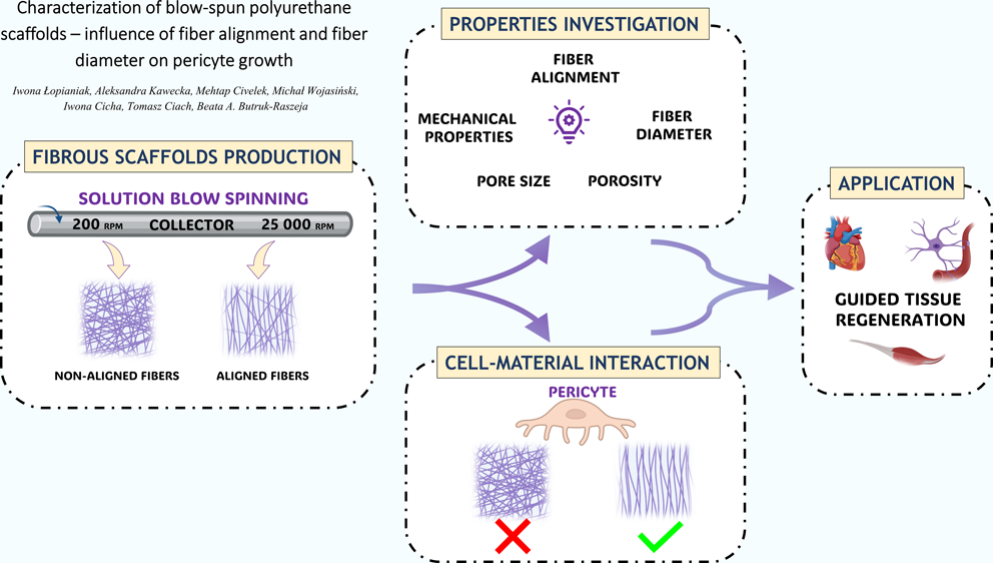

In this study, fibrous polyurethane (PU) materials with average fiber diameter of 200, 500, and 1000 nm were produced using a solution blow spinning (SBS) process. The effects of the rotation speed of the collector (in the range of 200–25 000 rpm) on the fiber alignment and diameter were investigated. The results showed that fiber alignment was influenced by the rotation speed of the collector, and such alignment was possible when the fiber diameter was within a specific range. Homogeneously oriented fibers were obtained only for a fiber diameter ≥500 nm. Moreover, the changes in fiber orientation and fiber diameter (resulting from changes in the rotation speed of the collector) were more noticeable for materials with an average fiber diameter of 1000 nm in comparison to 500 nm, which suggests that the larger the fiber diameter, the better the controlled architectures that can be obtained. The porosity of the produced scaffolds was about 65–70%, except for materials with a fiber diameter of 1000 nm and aligned fibers, which had a higher porosity (76%). Thus, the scaffold pore size increased with increasing fiber diameter but decreased with increasing fiber alignment. The mechanical properties of fibrous materials strongly depend on the direction of stretching, whereby the fiber orientation influences the mechanical strength only for materials with a fiber diameter of 1000 nm. Furthermore, the fiber diameter and alignment affected the pericyte growth. Significant differences in cell growth were observed after 7 days of cell culture between materials with a fiber diameter of 1000 nm (cell coverage 96–99%) and those with a fiber diameter of 500 nm (cell coverage 70–90%). By appropriately setting the SBS process parameters, scaffolds can be easily adapted to the cell requirements, which is of great importance in producing complex 3D structures for guided tissue regeneration.

## Introduction

The main role of scaffolds in tissue engineering is to provide structural support for cells, facilitate cell proliferation, sustain the mechanical properties of the replaced tissue, and create space for tissue regeneration. Due to their complex role, scaffolds are required to meet specific architectural and mechanical demands.^1−3^ Among the various scaffolds that can be selected for tissue engineering applications, fibrous scaffolds have attracted the attention of researchers owing to their unique topographical features. Fibrous constructs resemble the topography of an extracellular matrix (ECM), which provides a native microenvironment for cell adhesion, proliferation, differentiation, and migration.^1,4−6^ Furthermore, the ECM maintains the structure of the tissue and ensures its mechanical resistance, which is especially important when replacing tissues exposed to high forces, such as blood vessels. The ECM of blood vessels is formed by structural proteins (mainly collagens, elastin, and glycoproteins), the contents of which vary depending on the type of vessel. In addition to proteins, the vascular ECM constitutes a depot of resident growth factors and cytokines that regulate cell behavior.^7−12^

Pericytes play crucial roles in angiogenesis and vascular development. Together with endothelial cells (ECs) and smooth muscle cells (SMCs) they build blood vessel walls and ensure proper morphogenesis and homeostasis.^13^ Many layers of SMCs that build the tunica media are circumferentially wrapped around the ECs monolayer, providing vessel stability and regulating the blood flow. In contrast to SMCs, pericytes are directly embedded in the basement membranes of smaller vessels. Direct contact with ECs plays a crucial role in maintaining barrier function, cell–cell communication, and signal transmission along the length of the blood vessel.^14−16^ The complex and diverse characteristics of pericytes simultaneously stabilize hemodynamic processes, transmit signals along the vessel, and regulate blood flow, making them multifunctional and extremely important components of the circulatory system.

As each type of cell has different requirements regarding the matrix architecture, designing a suitable structure of scaffold is crucial to provide appropriate conditions for tissue reconstruction.^1,3,17^ The type of bulk polymer from which the scaffolds are made also influences their properties. Due to their high mechanical strength and degradation rate, PUs are attractive materials for tissue engineering applications, especially in vascular graft designing,.^18−20^ Solution blow spinning (SBS) is an emerging technique for fiber production that allows the fabrication of morphologically various well-controlled fibrous architectures that are increasingly used in tissue engineering.^21^ The properties of fibrous materials produced using this method can be easily customized to meet the requirements of tissue engineering applications.^22,23^ Heart, bone, skin, and vascular scaffolds have been widely produced using SBS.^20,24−29^

Several studies have been performed to define the influence of SBS process parameters, such as the polymer solution concentration, gas pressure, polymer solution flow rate, rotation speed of the collector, working distance, and nozzle design, on the fiber and fibrous material properties.^30,31^ It was concluded that there are dependent characteristics for each parameter (e.g., solution concentration influences fiber diameter and pore size,^32^ increasing the collector rotational speed results in fiber orientation,^33^ and reducing the working distance reduces the porosity of the material^28^), although the final properties of the fibrous scaffold result from the utilized polymer and the overall combination of all process parameters.

In this study, three types of fibrous PU materials with different fiber diameters (200, 500, and 1000 nm) were produced using the SBS method. Each of them was manufactured at rotation speeds of 200, 400, 1000, 5000, 10 000, 15 000, 20 000, and 25 000 rpm while maintaining the remaining process parameters constant. The main goal was to provide a comprehensive assessment of the influence of the rotation speed of the collector in the SBS process on PU fiber alignment depending on the fiber diameter as well as to evaluate the effect of the change in fiber alignment on the fiber diameter. Furthermore, the pore size, porosity, and mechanical properties of the fibrous materials with aligned and nonaligned fibers were compared, and the influence of fiber alignment and diameter on pericyte growth was evaluated.

## Materials and Methods

### 2.1 Polymer Solutions Preparation and Fibrous Scaffold Fabrication

The fibrous PU materials were produced from medical-grade PU ChronoFlexC75A (AdvanSource Biomaterials). To prepare polymer solutions, bulk PU was dissolved overnight in 1,1,1,3,3,3-hexafluoro-2-popanol (>99.0%, TCI Chemicals) using a magnetic stirrer at 25 °C. In this study, fibers were produced from PU solutions with concentrations of 2, 4, and 5% (weight/weight). The given concentrations were chosen to obtain materials with average fiber diameters of approximately 200, 500, and 1000 nm. Fibrous materials were produced using the SBS method as described in detail elsewhere.^32,34^ Briefly, PU solutions were placed in syringes and sprayed onto a rotating collector by using a concentric nozzle system. The nozzle system consisted of an inner nozzle with an inner diameter of 1.1 mm and an outer nozzle with an inner diameter of 4 mm. The nozzle lengths were 25 and 23 mm, respectively. The tip of the inner nozzle was protruded ahead of the tip of the outer nozzle by 2 mm. The polymer solution supplied through the inner nozzle is spun using a stream of compressed air supplied through the outer nozzle of the concentric nozzle system. The compressed air draws out the polymer solution from the inner nozzle and directs the polymer stream toward the rotating collector. The collector was mounted on the specific holder to move back and forward perpendicular to the fiber’s production direction, and the collector rotation was driven by an electric motor. A simple brush electric motor with a rotational speed in a range of 100–2000 rpm and with an operating voltage range of 1–7 V was used for production materials with a rotation speed of the collector 200–1000 rpm and brush electric motor for car models (Rally special 3, 17T super racer) with rotational speed up to 29 300 rpm and with operating voltage range of 3.7–9.6 V was used for production of the materials with a rotation speed of the collector 5000–25 000 rpm. Both motors were mounted on the same holder and connected to the collector pin. An appropriate nozzle-collector distance allows the solvent to evaporate from the polymer solution and collect fibers on the collector.

All materials were produced using the following process parameters: polymer solution flow rate of 30 mL/h, air pressure of 0.1 MPa, and working distance (between nozzle system and collector) of 30 cm for 120 min (200 nm), 30 min (500 nm), or 20 min (1000 nm) to obtain materials with a thickness of 300 μm. To investigate the influence of the rotation speed of the collector on fiber alignment, fibrous materials were produced at eight different rotation speeds: 200, 400, 1000, 5000, 10 000, 15 000, 20 000, and 25 000 rpm. The materials were collected on a cylindrical collector with a diameter of 12 mm, cut open, and analyzed as flat samples.

### 2.2 Scaffolds Characterization

#### 2.2.1 SEM Analysis

**Figure 1:**
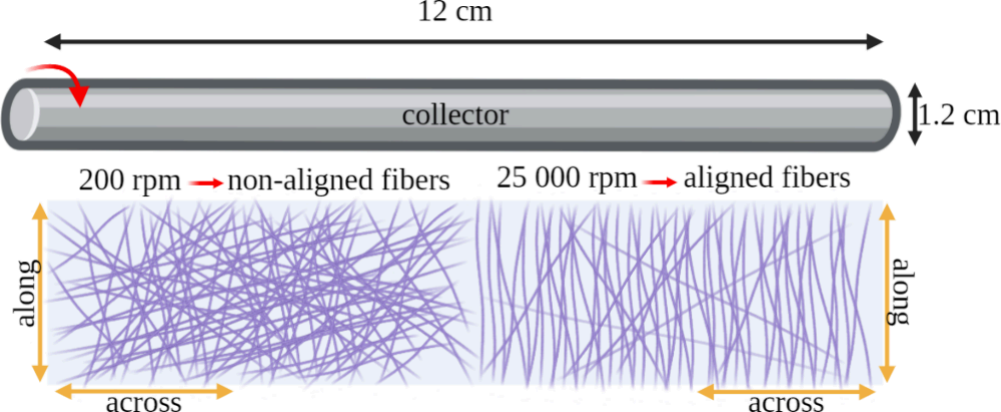
Schematic diagram of fibrous materials with nonaligned and aligned fibers and directions for analysis and measurements. Created in BioRender.com.

Material surface analysis was performed using scanning electron microscopy (SEM) (Phenom G1, Phenom World). To measure the fiber diameter, pore size, and fiber alignment, square samples with dimensions of 5 × 5 mm were glued to SEM stubs with conductive carbon adhesive tape and covered with a 15 nm thick gold layer using a sputter coater (K550 Emitech, Quorum Technologies). The samples were analyzed along the direction of wrapping the fibers on the collector, which was simultaneously the fiber alignment direction for the aligned materials (Figure 1). Images were taken at 600×, 1000×, and 5000× magnification. To perform the material thickness analysis, samples with dimensions of 10 × 10 mm were glued upright to SEM stubs with conductive carbon adhesive tape and covered with a 15 nm thick gold layer using a sputter coater. Images were taken at 300× magnification.

#### 2.2.2 Fiber Diameter and Pore Size

SEM images at 5000× magnification were used to measure the fiber diameter and pore area. The *n* = 100 fiber diameters and *n* = 100 pore areas of each sample were measured using the Fiji (ImageJ) software.^35^ The diameter of each analyzed fiber was measured using the Straight tool. Only the fiber diameters in the SEM images in the foreground were measured. The fiber diameter measurement results are presented as the average fiber diameter ± SD. To measure pore areas, the pores in the foreground of the SEM images were visualized with a Threshold tool and then the pore surfaces were measured with a Wand tool. To determine the pore size, the circular shape of the pores was assumed. The pore area (*A*_i_) measurement results were used to determine the pore size (*d*_p_) according to the following equation:
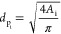
1The pore size measurement results are presented as average pore size ± SD.

#### 2.2.3 Fiber Alignment

SEM images at 1000× magnification were used to determine the fiber alignment depending on the rotational speed of the collector in each material. For this purpose, *n* = 150 fiber angle deviations from the alignment direction were measured using Fiji (ImageJ) software. A line perpendicular to the bottom edge of the image was assumed to be the alignment direction. The angles between fiber and alignment direction line were measured using a Straight tool. It was presumed that an angle deviation of <30° indicated preferably oriented (aligned) fibers. The results are presented as the average fiber deviation angle ± SD.

#### 2.2.4 Material porosity

The gravimetric method was used to determine the material porosity. For each type of analyzed material, *n* = 3 samples with dimensions of 10 × 10 mm were weighed on an analytical balance to determine the sample mass (m_i_. Photographs of the samples were taken, and their surface areas (*A*_i_) were determined using Fiji (ImageJ) software. The SEM images (*n* = 5) of each sample cross-section at 300× magnification were used to determine the sample thickness. Subsequently, *n* = 6 thickness measurements were performed for each SEM image and the average thickness (δ_i_) was determined. The scaffold density (ρ_s_i__) was calculated by using the following formula:
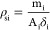
2The porosity (ε) was determined according to the equation:
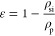
3where the polymer density is ρ_p_ = 1.2 g/cm^3^.^36^ The results are presented as the average material porosity ± SD.

#### 2.2.5 Mechanical Properties

A static tensile test was performed using an Instron3345 instrument equipped with a 50 N static load cell. The materials were subjected to mechanical tests along and across the direction of wrapping the fibers on the collector as presented in Figure 1. Rectangular samples with dimensions of 70 × 5 mm (*n* = 5) were cut from each material in both directions and placed in the pneumatic jaws of the Instron machine. The distance between the jaws and the initial sample length was set at 5 cm. The samples were stretched at a rate of 5 mm/min until they broke. Dedicated Bluehill software automatically determined the Young’s modulus, elongation at break, and maximum tensile stress for each sample. The results are presented as average values ± SD.

### 2.3 Pericytes Culture and Seeding

Human pericytes from placenta tissue (hPC–PL, Promocell, Germany) were thawed according to the manufacturers’ instructions and cultured in supplemented pericyte growth medium (Pericyte Growth Medium 2, Promocell, Germany) at 37 °C in a 5% CO_2_ humidified atmosphere, in cell 75 cm^3^ cell culture flasks (TPP Techno Plastic Products AG, Switzerland). The medium was changed every 2 days. Accutase solution (Promocell, Germany) was used to harvest cells. Cells at passage 5 were used in the experiments. The experiment was repeated 3 times.

For each type of analyzed material, *n* = 2 round samples were sterilized with 70% ethanol for 20 min and washed with sterile PBS (3 × 5 min). Sterile samples were mounted on cell culture inserts (Scaffdex, Sigma-Aldrich, Munich, Germany) and placed in 48-well plates. Before cell seeding, the samples were incubated in the medium for 1 h at 37 °C. Then, a pericyte suspension in growth medium (3 × 10^4^ cells/sample) was added to each well with the sample. Well plates were incubated at 37 °C in a humidified atmosphere containing 5% CO2 for 1, 3, and 7 days. Culture media were changed 24 h after seeding and every second day thereafter.

### 2.4 Cell Adhesion Analysis

After each culture period, cells growing on fibrous samples were fixed at 4 °C for 15 min in 4% buffered paraformaldehyde (Roth GmbH, Karlsruhe, Germany). Afterward, the samples were washed with PBS (3 × 5 min), and the cells were permeabilized with 0.2% Triton X-100 (Sigma-Aldrich) in PBS (8 min). After washing with PBS (3 × 5 min), a staining solution containing 1% Alexa488-phalloidin (Invitrogen, Thermo Fisher) and 0.1% Hoechst 33342 (Invitrogen, Thermo Fisher) was used to stain F-actin filaments and nuclei, respectively. Finally, the samples were washed with PBS (3 × 5 min) and prepared for fluorescence imaging.

Images of each sample (*n* = 5 images per sample) at 10× magnification were obtained using a Zeiss Axio Observer Z1 fluorescence microscope (Zeiss, Jena, Germany). Cell coverage was measured on each image using Fiji (ImageJ) software. The results are presented as the average cell coverage, ± SD.

### 2.5 Statistical Analysis

The statistical significance of the differences was analyzed using single-factor analysis of variance (ANOVA) for *p* ≤ 0.05, with posthoc Tukey’s test (OriginPRO 2021b).

## Results

### 3.1 Fiber Alignment

**Figure 2:**
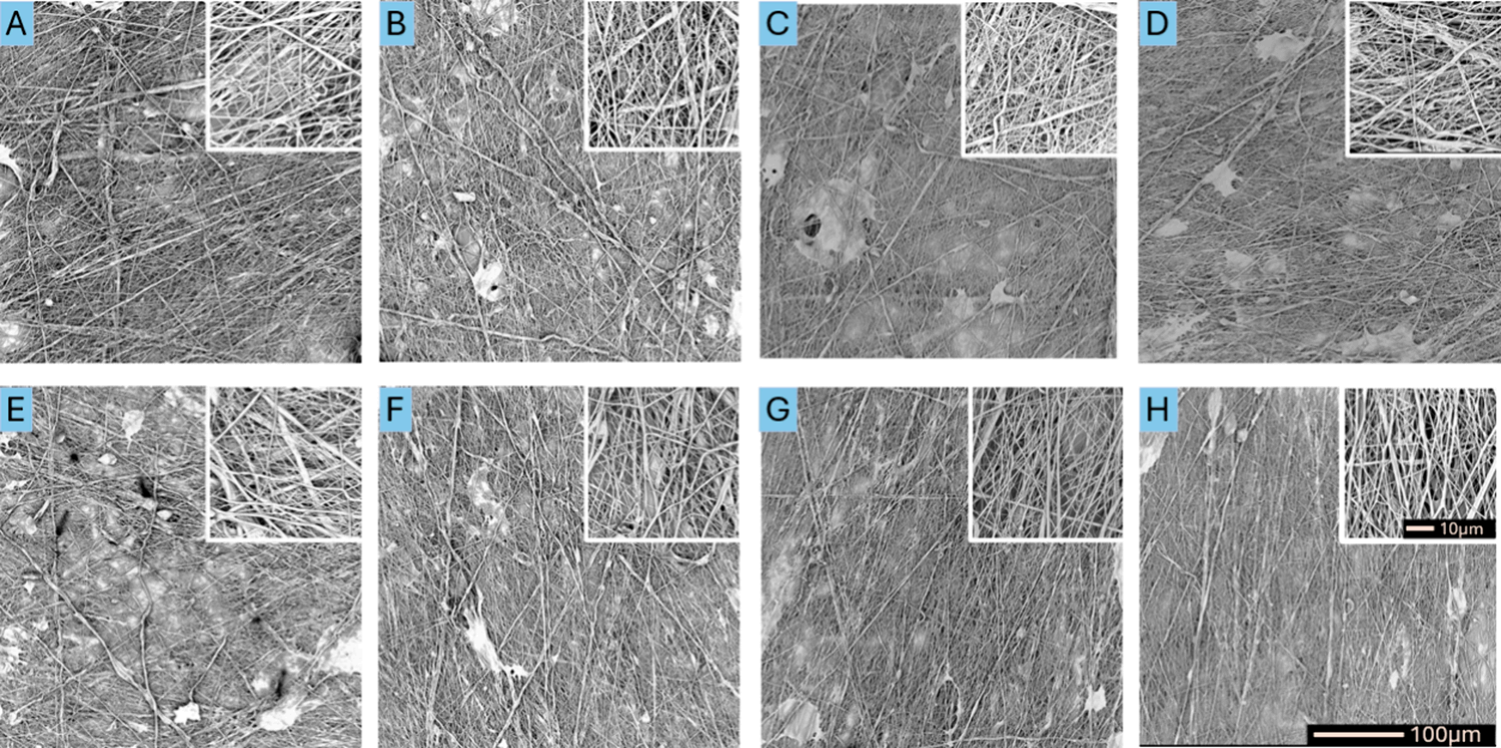
SEM images of fibrous materials with an average diameter of 200 nm produced at collector rotation speeds of (A) 200 rpm, (B) 400 rpm, (C) 1000 rpm, (D) 5000 rpm, (E) 10 000 rpm, (F) 15 000 rpm, (G) 20 000 rpm, and (H) 25 000 rpm at a magnification of 600× and 5000×.

**Figure 3:**
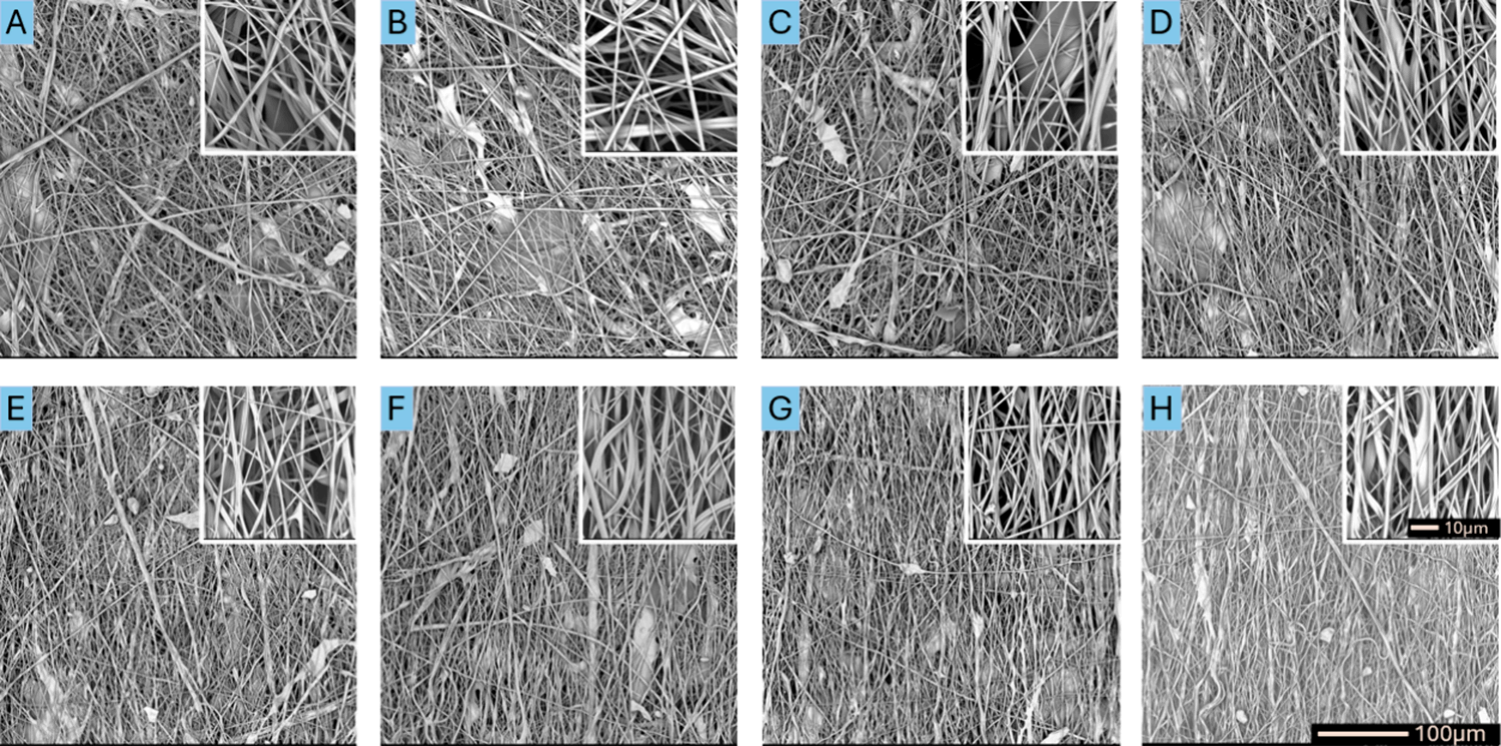
SEM images of fibrous materials with an average diameter of 500 nm produced at collector rotation speeds of (A) 200 rpm, (B) 400, (C) 1000, (D) 5000, (E) 10 000, (F) 15 000, (G) 20 000, and (H) 25 000 rpm at a magnification of 600× and 5000×.

**Figure 4:**
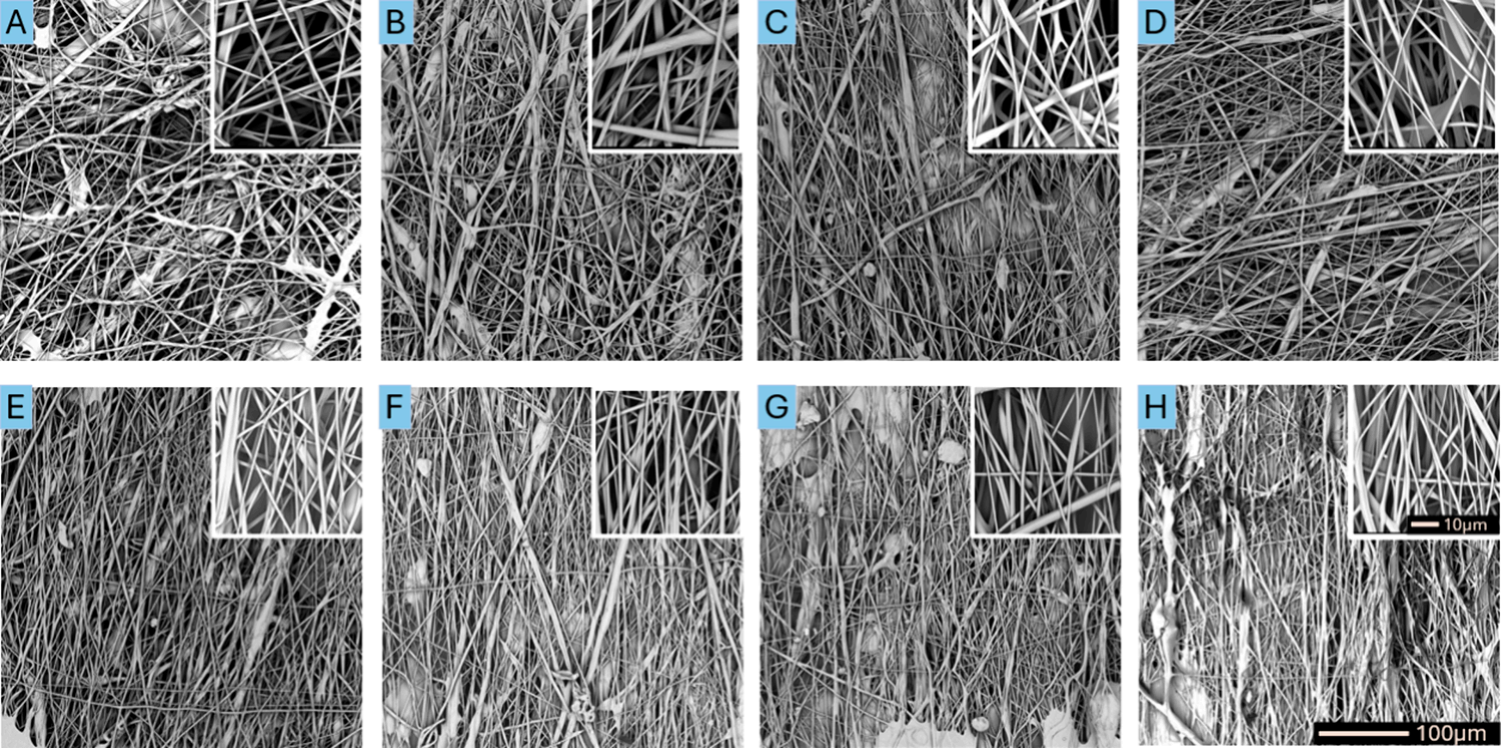
SEM images of fibrous materials with an average diameter of 1000 nm produced at collector rotation speeds of (A) 200 rpm, (B) 400 rpm, (C) 1000 rpm, (D) 5000 rpm, (E) 10 000 rpm, (F) 15 000 rpm, (G) 20 000 rpm, and (H) 25 000 rpm at a magnification of 600× and 5000×.

**Figure 5:**
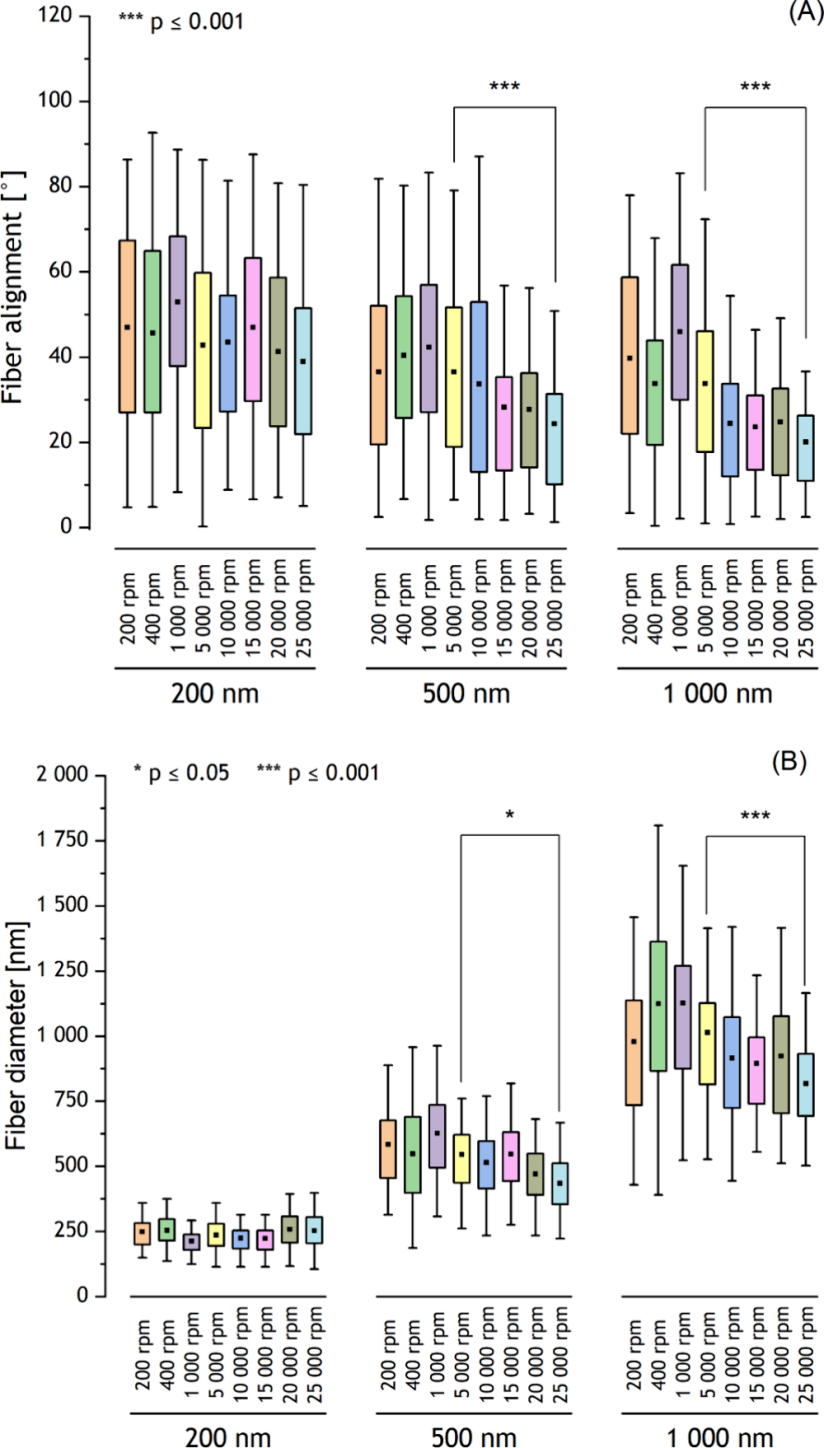
(A) Fiber alignment, AVR ± SD, 150; (B) fiber diameter, AVR ± SD, 100; the square in the middle of the box of the box plot indicates the mean value, the box indicates the interquartile range (IQR) (25th–75th percentile), and the whiskers indicate the range within 1.5IQR (5th–95th percentile). For materials with an average fiber diameter of 200 nm the change of collector rotational speed did not significantly influence an average fiber alignment as well as fiber diameter. However, there is a remarkable difference in the fiber alignment obtained for 5000 rpm and 25 000 rpm for materials with an average fiber diameter of 500 nm and 1000 nm (*p* ≤ 0.001). Furthermore, for materials with an average fiber diameter of 500 nm and 1000 nm significant decrease in fiber diameter was noticed, whereas a more significant decrease was observed for materials with an average fiber diameter of 1000 nm (*p* ≤ 0.05 and *p* ≤ 0.001 for materials with an average fiber diameter 500 and 1000 nm, respectively).

First part of this study, the influence of the rotational speed of the collector on the fiber alignment of materials with different average fiber diameters was evaluated. SEM images of fibrous materials with average fiber diameters of 200, 500 and 1000 nm, produced with different collector rotational speeds (200, 400, 1000, 5000, 10 000, 15 000, 20 000, and 25 000 rpm) are presented in Figures 2–4. Additionally, in Figure 5(A), the results of the fiber alignment measurements are shown.

Representative SEM images of materials surface morphology (Figures 2–4) show typical morphology of fibrous PU materials produced by SBS method, characterized by the presence of fibers and single defects in the form of stains.^28,32,34^ There were no significant differences in the fiber alignment, depending on the rotation speed of the collector in materials with an average fiber diameter of 200 nm (Figure 2). Differences in fiber alignment appeared in the SEM images of the materials with average fiber diameters of 500 and 1000 nm. At low rotation speeds (200, 400, 1000, and 5000 rpm), the fibers were randomly oriented regardless of the average fiber size. However, with an increase in the rotation speed to 10 000, or 15 000 rpm, a more uniform arrangement of fibers was observed, whereas at rotation speeds of 20 000 and 25 000 rpm, homogeneously oriented (aligned) fibers were visible (Figures 3 and 4).

The results of the qualitative analysis of the surface of each material were quantitatively confirmed by measuring the fiber deviation angles from the preferred orientation direction (Table 1, Figure 5(A)). The deviation angles obtained for materials with an average fiber diameter of 200 nm were about 40–50° regardless of the rotation speed of the collector. The fiber deviation angles obtained for materials with average fiber diameters of 500 nm and 1000 nm produced at rotation speeds in the range of 200–5 000 rpm were in the range of 30–45°, whereas increasing the rotation speed above 10 000 rpm allowed us to obtain deviation angles below 30°. Lower deviation angles were obtained for materials with an average fiber diameter of 1000 nm in comparison with 500 nm for the same rotation speed of the collector.

**Table 1:** Results of Fiber Alignment and Fiber Diameter Measurements (AVR ± SD)

	Deviation angle (deg)			Fiber diameter (nm)		
**Rotation speed of collector (rpm)**	**200 nm**	**500 nm**	**1000 nm**	**200 nm**	**500 nm**	**1000 nm**
200	47 ± 23	37 ± 21	40 ± 21	250 ± 58	585 ± 193	979 ± 337
400	46 ± 22	40 ± 18	34 ± 18	255 ± 63	548 ± 185	1126 ± 370
1000	53 ± 20	42 ± 20	46 ± 20	214 ± 45	628 ± 195	1127 ± 370
5000	43 ± 22	37 ± 19	34 ± 19	236 ± 68	546 ± 160	1014 ± 305
10 000	44 ± 20	34 ± 24	24 ± 16	225 ± 65	514 ± 167	916 ± 278
15 000	47 ± 21	28 ± 19	24 ± 15	223 ± 65	547 ± 153	895 ± 254
20 000	41 ± 20	28 ± 18	25 ± 17	258 ± 72	471 ± 129	924 ± 296
25 000	39 ± 19	24 ± 18	20 ± 13	253 ± 79	435 ± 116	818 ± 217

The fiber deviation angle distributions in the form of histograms are presented in Figure S1. The graphs show a noticeable change in the fiber alignment for materials with an average diameter of 500 and 1000 nm and no change for materials with an average fiber diameter of 200 nm. For the analyzed materials with diameters of 500 nm and 1000 nm, the change in fiber alignment occurred when the rotation speed of the collector was 10 000 rpm or more. Statistical analysis showed no significant differences in the fiber alignment for materials with an average fiber diameter of 200 nm, regardless of the rotation speed. Furthermore, significant changes in the fiber alignment for materials with average fiber diameters of 500 and 1000 nm produced at rotation speed 5000 rpm and 25 000 rpm (*p* ≤ 0.001) were observed. A fiber deviation angle <30° indicating aligned (homogeneously oriented) fibers has been achieved at a rotational speed ≥15 000 rpm for materials with an average fiber diameter of 500 nm, and ≥10 000 rpm for materials with an average fiber diameter of 1000 nm. Thus, in further analysis, materials with average fiber diameters of 500 nm and 1000 nm produced at rotation speed of 5000 and 25 000 rpm were considered as nonaligned and aligned, respectively.

Additionally, in Figure S2 the results of fiber thickness measurements depending on the rotation speed of the collector are shown. Moreover, representative SEM images used for material thickness determination are presented in Figure S3. The average thickness of the materials is in the range of 250–350 μm regardless of the collector rotation speed.

### 3.2 Fiber Diameter

The results of the evaluation of the influence of the rotation speed of the collector on the fiber diameter are presented in Figure 5(B). For materials with an average fiber diameter of 200 nm, no significant differences in the fiber diameter were observed, whereas for materials with average fiber diameters of 500 and 1000 nm, a slight decrease in the fiber diameter was observed with an increase in the rotational speed of the collector. The results of the fiber diameter measurements are presented in Table 1.

The fiber diameter distributions in the form of histograms are presented in Figure S4. For materials with average diameters of 500 and 1000 nm, the distributions became narrower as the rotation speed of the collector increased. Moreover, a slight shift in the distributions toward smaller diameters was observed. For materials with an average fiber diameter of 200 nm, no change in the fiber diameter distribution was observed regardless of the rotation speed. For materials with an average fiber diameter of 500 nm and 1000 nm, significant decrease in fiber diameter was noticed with increasing rotation speed, whereby a more significant decrease was observed for materials with an average fiber diameter of 1000 nm (*p* ≤ 0.05 and *p* ≤ 0.001 for materials with an average fiber diameter 500 and 1000 nm, respectively).

In the next part of this study, the properties of fibrous materials with diameters of 500 and 1000 nm with nonaligned (NA) and aligned (A) fibers were compared. The materials were produced with collector rotational speeds of 5000 and 25 000 rpm for nonaligned and aligned fibers and marked as 500_NA, 500_A, 1 000_NA, and 1 000_A, respectively.

### 3.3 Porosity and Pore Size

**Figure 6:**
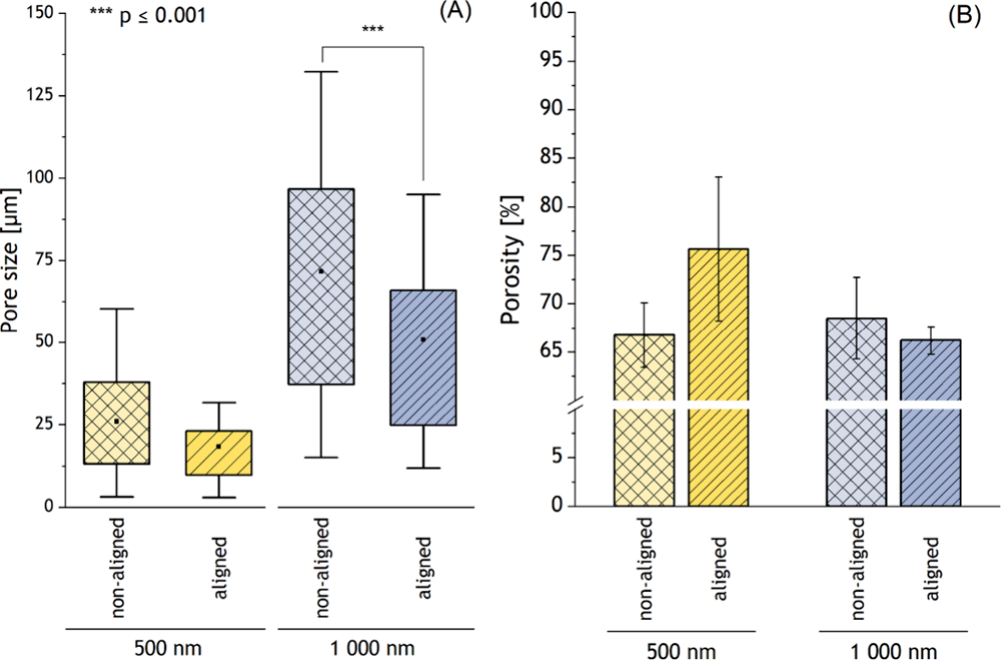
Scaffold (A) pore size and (B) porosity depending on the fiber diameter and alignment, AVR ± SD, *n* = 50; the square in the middle of the box of the box plot indicates the mean value, the box indicates the interquartile range (IQR) (25th–75th percentile), and the whiskers indicate the range within 1.5IQR (5th–95th percentile). An increase in pore size value was observed only for aligned fibers with an average fiber diameter of 1000 nm (*p* ≤ 0.001). There were no significant changes in material porosity regardless of fiber diameter and alignment.

The results of the pore size and porosity measurements are shown in Figure 6 and Table 2. The pore size (Figure 6(A)) of the fibrous scaffolds increased with the fiber diameter. Moreover, for materials with an average diameter of 1000 nm, fiber alignment influenced the pore size. For scaffolds with aligned fibers (*d* = 1000 nm), the pore size values were significantly lower (*p* ≤ 0.001). No significant changes in the pore size were observed for the materials with an average fiber diameter of 500 nm. Moreover, there were no significant changes in material porosity (Figure 6(B)). The average porosity of the scaffold was >65%, regardless of fiber diameter and alignment.

**Table 2:** Results of Pore Size, Porosity, and Mechanical Parameters Measurements (AVR ± SD)

		**Sample**			
		**500_NA**	**500_A**	**1 000_NA**	**1 000_A**
**Pore size (μm)**		26 ± 15	18 ± 11	72 ± 45	51 ± 40
**Porosity (%)**		67 ± 3	76 ± 6	69 ± 4	66 ± 1
**Young’s modulus (MPa)**	**along**	6.6 ± 2.0	6.5 ± 0.8	4.3 ± 1.1	7.5 ± 0.5
	**across**	1.7 ± 0.7	0.8 ± 0.0	0.8 ± 0.2	0.8 ± 0.1
**Elongation at break (%)**	**along**	123 ± 23	85 ± 36	143 ± 28	141 ± 30
	**across**	216 ± 39	215 ± 11	250 ± 49	227 ± 11
**Maximum tensile stress (MPa)**	**along**	14.1 ± 2.0	12.3 ± 5.3	13.4 ± 1.1	20.3 ± 4.2
	**across**	8.0 ± 0.4	5.5 ± 0.3	5.4 ± 0.4	4.8 ± 0.3

### 3.4 Mechanical Properties

**Figure 7:**
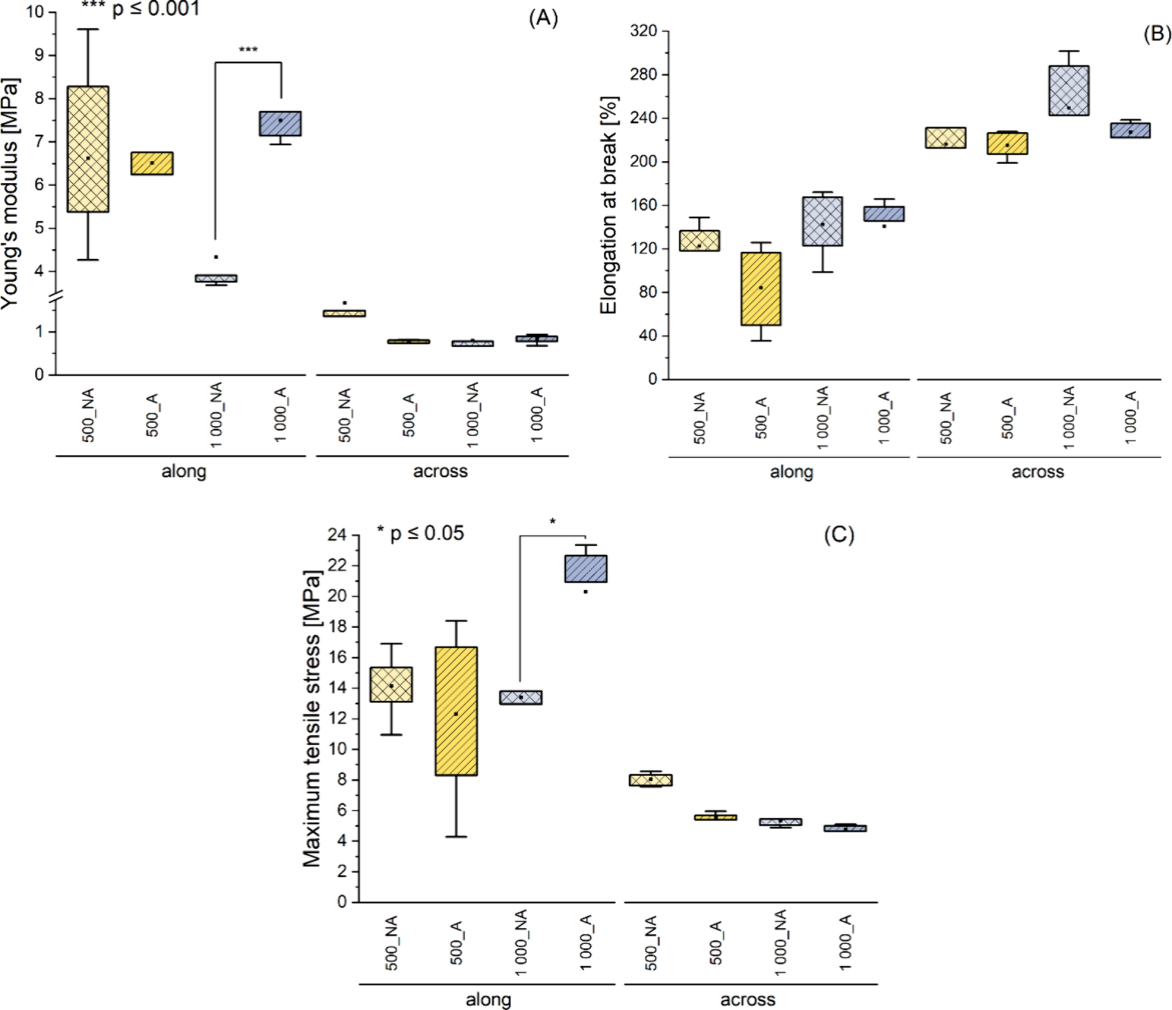
Mechanical properties of fibrous samples with diameters 500 and 1000 nm with nonaligned and aligned fibers subjected to static tensile test along and across fibers alignment direction; (A) Young’s modulus, (B) maximum force, (C) maximum tensile stress, AVR ± SD, *n* = 5; the square in the middle of the box of the box plot indicates the mean value, the box indicates the interquartile range (IQR) (25th–75th percentile), and the whiskers indicate the range within 1.5IQR (5th–95th percentile). For materials with an average fiber diameter of 1000 nm stretched in a along direction, a significant reduction in Young’s modulus value was observed for samples with nonaligned fibers in comparison to aligned (*p* ≤ 0.001); Samples elongation at break values were greater for more elastic materials (lower YM values), however, there were no meaningful differences regardless of stretching direction, fiber diameter, or alignment. For samples stretched in the along direction, a significant increase (*p* ≤ 0.05) of maximum tensile stress was observed for aligned materials with an average fiber diameter of 1000 nm.

Fibrous materials were subjected to static tensile tests in two directions: along and across the direction of wrapping the fibers on the collector, hereinafter referred to as directions: along and across. The results presented in Figure 7 indicate distinct differences in the mechanical properties of the samples depending on the stretching direction. The measured mean values of the mechanical properties of the materials are listed in Table 2.

The Young’s modulus (YM) measurement (Figure 7(A)) showed a significant difference in material elasticity depending on the stretching direction. The YM values obtained for materials stretched across the direction of wrapping fibers on the collector (independent of fiber alignment and diameter) are decidedly lower than those obtained for materials stretched along. Moreover, a significant reduction in the YM value was observed for samples with nonaligned fibers in comparison to aligned fibers (*p* ≤ 0.001) only for materials with an average fiber diameter of 1000 nm stretched along. In the remaining variants, the fiber alignment did not significantly affect the elasticity of the material.

The elongation at break values (Figure 7(B)) was greater for more elastic materials (lower YM values), but no meaningful differences were observed regardless of the stretching direction, fiber diameter, or alignment.

The maximum tensile stress values (Figure 7(C)) were higher for materials stretched along regardless of the fiber diameter and alignment. In these samples, a significant increase (*p* ≤ 0.05) in the maximum tensile stress was observed for the aligned materials with an average fiber diameter of 1000 nm.

### 3.5 Cellular Response

**Figure 8:**
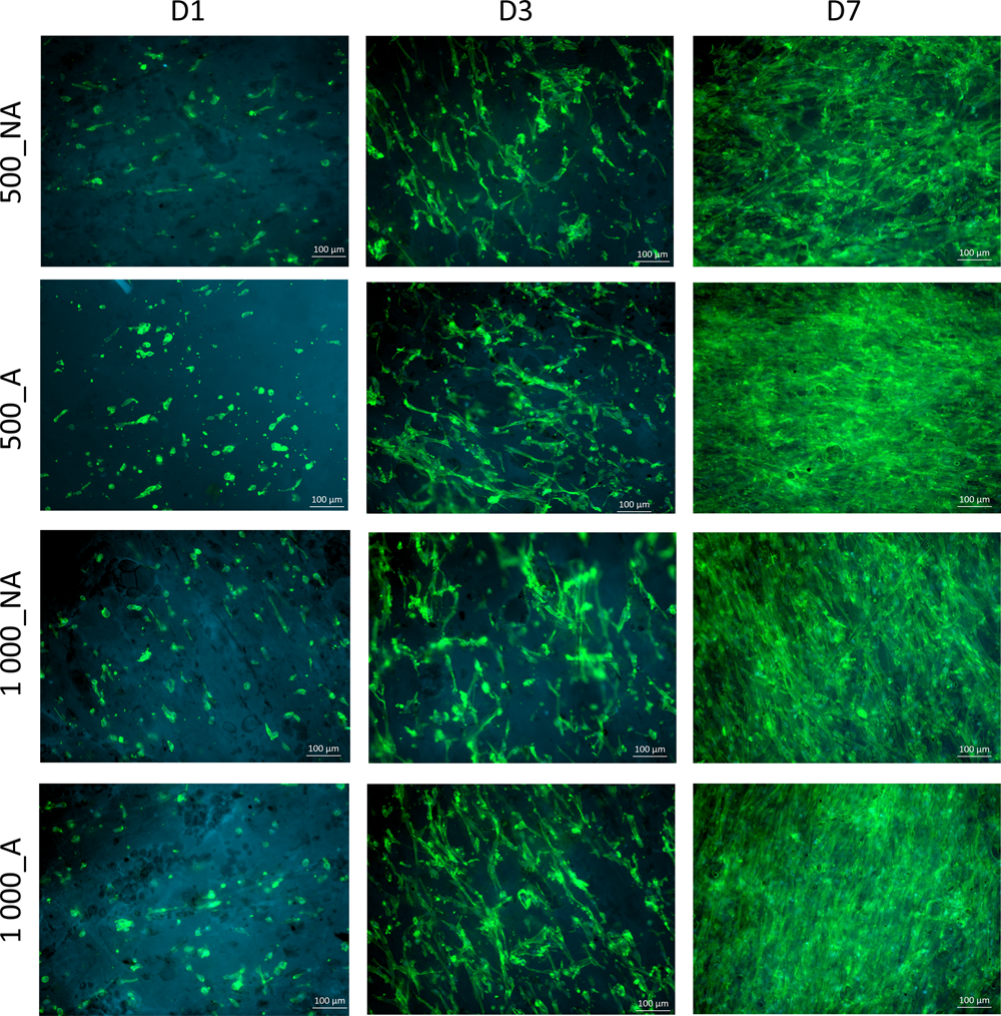
Pericyte adhesion on aligned (A) and nonaligned (NA) fibers of materials with an average fiber diameter of 500 and 1000 nm, after 1, 3, and 7 days in magnification 10×.

**Figure 9:**
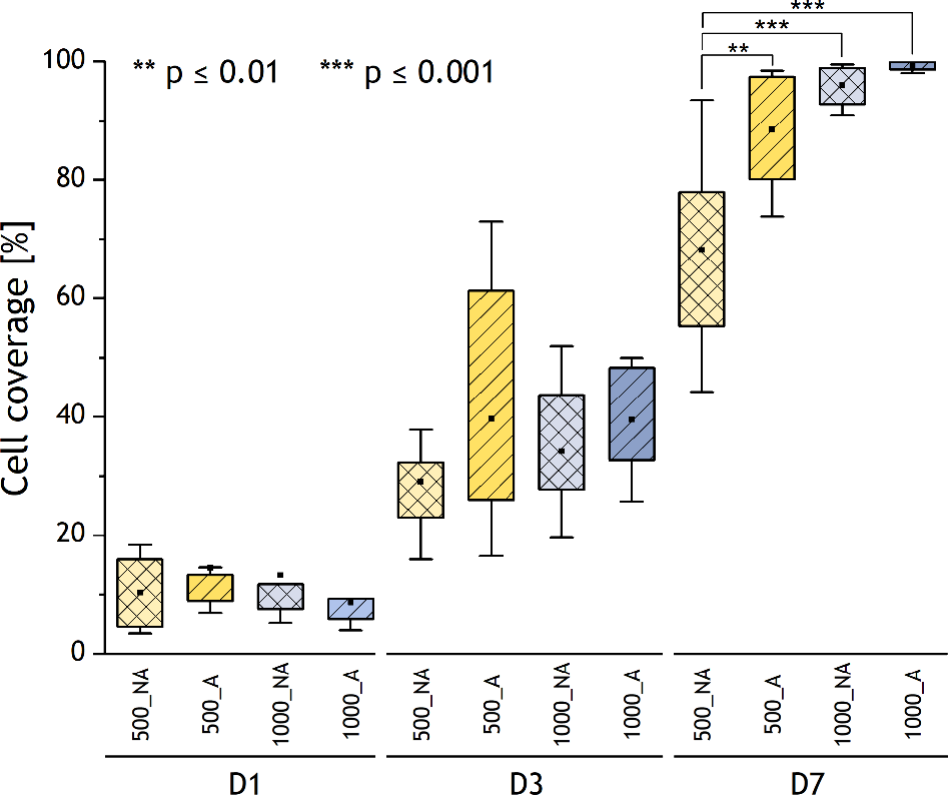
Pericyte coverage of the analyzed materials; the square in the middle of the box of the box plot indicates the mean value, the box indicates the interquartile range (IQR) (25th–75th percentile), and the whiskers indicate the range within 1.5IQR (5th–95th percentile). Cell coverage after 1 day of cell culture was similar for all analyzed materials; There were no significant differences in cell coverage after 1 and 3 days of cell culture, regardless of the type of material. The cell coverage (after 7 days of cell culture) was ≥89% for aligned materials with a diameter of 500 nm and aligned fibers and 1000 nm for both aligned and nonaligned samples. There was a notably lower cell coverage (68%) for nonaligned materials with fiber diameter of 500 nm compared to other types of analyzed materials (*p* ≤ 0.01 and *p* ≤ 0.001).

To investigate cell-material interactions depending on fiber diameter and alignment, pericytes were cultured on nonaligned and aligned materials with fiber diameters of 500 and 1000 nm. Images of the cells growing on the respective materials after 1, 3, and 7 days are presented in Figure 8. The cell-coverage measurement results are presented in Figure 9 and Table 3.

**Table 3:** Results of Cell Coverage Measurements, AVR ± SD

	**Cell coverage (%)**			
	**500_NA**	**500_A**	**1 000_NA**	**1 000_A**
**Day 1**	10 ± 5	14 ± 13	13 ± 11	10 ± 4
**Day 3**	29 ± 10	40 ± 19	34 ± 10	40 ± 8
**Day 7**	68 ± 15	89 ± 9	96 ± 3	99 ± 1

At day 1 postseeding, pericytes attached to aligned materials with an average fiber diameter of 500 nm were more elongated than cells attached to aligned materials with an average fiber diameter of 1000 nm. Cell coverage after 1 day of cell culture was similar for all of the analyzed materials. After 3 days of cell culture, pericytes growing on aligned materials (500_A, 1 000_A) were more elongated in shape than cells growing on nonaligned fibers (500_NA, 1 000_NA), and the cell coverage was likewise slightly higher for aligned materials. There were no significant differences in cell coverage after 1 and 3 days of cell culture, regardless of the type of material. Independent of fiber diameter, after 7 days of cell culture, pericytes were elongated, homogeneously oriented, and formed a firm layer on the aligned materials. In contrast, some empty areas that were not fully colonized by cells were visible on the nonaligned materials. Moreover, on nonaligned samples, pericytes were randomly oriented and single, not fully elongated cells were observed. However, the cell coverage (after 7 days of cell culture) was similar (≥89%) for aligned materials with a fiber diameter of 500 nm and both aligned and nonaligned fibers with a diameter of 1000 nm. Moreover, there was notably lower cell coverage (68%) on nonaligned materials with fiber diameter of 500 nm compared to other types of analyzed materials (*p* ≤ 0.01 and *p* ≤ 0.001).

## 4 Discussion

Although the influence of process parameters on the properties of fibers produced by electrospinning has been extensively investigated,^37−41^ relatively little is known about how the SBS process parameters affect the characteristics of the produced fibers. Although electrospinning and SBS are similar methods, considerable process differences prevent direct comparisons or extrapolations between these methods.

In this study, a comprehensive assessment of the impact of the rotation speed of the collector during the SBS process on the fiber morphology and the physical and mechanical properties of the scaffold, depending on the fiber diameter, is presented. Furthermore, we evaluated the cell-material interactions depending on the fiber diameter and their alignment. The rotation speed of the collector is one of the SBS process parameters that affects fiber morphology. According to literature, uniformly arranged fibers are acquired in the SBS process by increasing the rotation speed of the collector.^33,42,43^ In our previous study, we evaluated the influence of the polymer solution concentration, compressed gas pressure, and polymer solution flow rate on the fiber diameter and number of defects on the scaffold surface.^32^ Here, we investigated the impact of the rotation speed of the collector on the PU fiber alignment with respect to the fiber diameter.

We successfully produced ∼300 μm thick fibrous materials with average fiber diameters of 200, 500, and 1000 nm using 8 different rotation speeds of collector. After evaluating the fiber alignment, we observed that the fiber diameter limits the possibility of obtaining parallel polyurethane fibers (Figure 2, Figure 5(A-B)). For materials with an average fiber diameter of 200 nm, no change in fiber alignment was observed with an increasing rotation speed, and the change in rotation speed did not influence the average fiber diameter. The fiber deviation from the alignment direction was 40–45° for all analyzed rotation speeds of the collector, which is characteristic of nonaligned, randomly distributed polyurethane fibrous materials produced by the SBS method. A similar effect was reported by Pimenta et al.,^20^ who produced poly(ε-caprolactone) (PCL) fibers with an average diameter of approximately 200 nm at rotation speeds of 200 and 750 rpm. The authors did not observe the influence of the rotation speed of the collector on the fiber diameter, and changing the rotation speed did not affect the fiber alignment. In contrast, Simbara et al.^33^ obtained aligned PCL fibers with an average diameter of approximately 300 nm at a rotational speed of 300 rpm. In general, there are only a few reports showing the modeling of fiber alignment in spinning processes such as solution blow spinning. Sinha-Ray et al. showed that by increasing the collector movement in their model, the fibers were collected on a moving screen, and the placement of the fibers became ordered.^44^ They confirmed their results further for a wider range of collector speeds and published them in a book.^45^ Thus, the presence of fiber alignment was partially predicted numerically in the work of Sinha-Ray et al. and Yarin et al., where the fiber sizes varied from 300 nm to 2 mm.

The results obtained in this study showed that increasing the rotation speed of the collector during the production of materials with an average fiber diameter of ≥500 nm resulted in a decrease in the average fiber deviation from the alignment direction values down to 20–25°. A significant change in the fiber alignment for materials with an average fiber diameter of 500 and 1000 nm was observed when the rotation speed was 25 000 rpm. According to the SEM images and fiber alignment measurements, materials with diameters ≥500 nm, produced at rotation speeds of 5000 and 25 000 rpm, were clearly distinguishable as nonaligned and aligned (homogeneously oriented), respectively. Moreover, the change in fiber orientation resulted in a slight decrease in the average fiber diameter values for materials with average fiber diameters of 500 and 1000 nm. A significant decrease in fiber diameter was noticed when the rotation speed was 25 000 rpm, whereas the change was more significant for materials with an average fiber diameter of 1000 nm. Additionally, fiber alignment as well as fiber diameter distributions for materials with average fiber diameters of 500 and 1000 nm became narrower when the rotation speed of the collector increased. The observed relationship may result from the fact that as the collector rotational speed increases the fibers are wound onto the collector faster, resulting in their stretching, which is observed as a decrease in diameter. Moreover, at higher rotational speeds, fibers with larger diameters may deposit on the collector worse due to greater inertia in relation to the centrifugal force of the collector.

Czarnecka et al. performed a correlative analysis of fiber size as a function of polymer concentration and rotational speed of the collector for PCL fibers produced in SBS.^46^ Although the slight correlation between fiber size and rotational speed is visible for the largest fibers (about 500 nm for polymer concentration of 9%w/w) appeared, and the mean fiber size drops slightly with rotational speed, the authors explicitly stated that “No significant influence of collector rotational speed on average fiber diameter was found”.^46^ Furthermore, as described by González-Benito et al.,^43^ thinner poly(ethylene oxide) fibers were produced by increasing the rotation speed of the collector. Additionally, their results showed that together with a decrease in the average fiber diameter, the fiber size homogeneity increases (diameter distribution narrows), which was also observed in this study (Figures S1 and S4). Variations in fiber orientation and diameter were more noticeable for materials with an average fiber diameter of 1000 nm in comparison to 500 nm, which suggests that the greater the fiber diameter, the better control over the produced architectures is achievable. The results confirmed that it is possible to obtain aligned fibers by the solution blow spinning. However, this is the preferred direction, not the ideal alignment.

Concerning the mechanical properties of fibrous scaffolds (Figure 8), the obtained results showed that they strongly depend on the direction of stretching, whereas fiber orientation influences the mechanical strength more strongly for materials with a fiber diameter of 1000 nm. The elasticity of scaffolds stretched in the along direction was lower than scaffolds stretched across (Young’s modulus values 4.3–7.5 and 0.8–1.7 MPa for samples stretched along and across fibers, respectively), while the opposite was observed for the mechanical strength. Fiber alignment significantly influences the mechanical properties of materials with an average fiber diameter of 1000 nm, stretched only along (parallel to aligned fibers). Aligned samples were less elastic (Young’s modulus values: 4.3 and 7.50 MPa for nonaligned and aligned fibers) but showed greater tensile strength than nonaligned materials (maximum tensile stress values were 13 and 20 MPa for aligned and nonaligned materials, respectively). For materials with an average fiber diameter of 500 nm stretched in both directions, the fiber alignment did not influence the mechanical strength. In our previous study,^47^ we observed similar dependences in Young’s modulus and tensile stress values for PLLA and PU nanofibers tested in two directions: parallel and perpendicular to fibers orientation. Moreover, Simbara et al.^33^ also reported an increase in stress values for samples stretched along the oriented fibers. These results suggest that the method of fiber formation has the greatest influence on the mechanical properties of the fibrous materials.

In our study, the fibers were wrapped around the collector, which means that regardless of the rotation speed, the topography of the scaffold was formed by fibers arranged more or less uniformly along the collector. The highest mechanical strength of the materials stretched parallel to the aligned fibers seems to result from a larger number of fibers arranged in this direction. Presumably, this larger number of fibers arranged in one direction reduced the ability of the material to return to its original shape, which was observed as a decrease in the elasticity of the material. The difference in the mechanical properties of the aligned and nonaligned scaffolds was only observed when the fiber diameter was 1000 nm, which confirms the previous conclusion that by increasing the fiber diameter, materials with a better-controlled architecture can be obtained. Simbara et al.^33^ compared the mechanical resistance of aligned and nonaligned fibrous scaffolds in two directions. Their results also suggested that material strength strictly depends on the direction of stretching, much more than on fiber alignment.

The properties of scaffolds should be adjusted according to the requirements of the tissue to be replaced.^48^ This study aimed to produce polyurethane (PUs) scaffolds for potential vascular engineering applications. The results of mechanical tests showed similarities to autologous vessels (e.g., the elastic modulus values of human arteries are 1–8 MPa^49,50^). The high porosity and adequate pore size of the scaffolds are other important factors that enable tissue reconstruction. In a study by Pimenta et al.,^20^ vascular scaffolds with a porosity of 50–75% and pore sizes of 7–30 μm were successfully populated with cells. The porosity of the produced scaffolds was about 65–70%, with the exception of materials with a fiber diameter of 1000 nm and aligned fibers, which had a higher porosity (76%). The scaffold pore size increased with increasing fiber diameter but decreased with increasing fiber alignment (26 μm for nonaligned versus 18 μm for aligned materials with a fiber diameter of 500 nm, and 72 μm for nonaligned and 50 μm for aligned materials with a fiber diameter of 1000 nm). This porosity range seemed appropriate for vascular regeneration.

In addition to providing mechanical support, the scaffold architecture is a topographic guide for cells.^51^ In vascular applications, the topography of the prosthesis should be layered to reproduce the structure of a native vessel, and the architecture of each layer should satisfy the requirements of distinct cell types.^52^ Pericytes are known to exhibit characteristics similar to SMC and play an important role in blood vessel formation,^14,15^ but their structural demands for effective scaffold colonization are not fully known. Therefore, we evaluated the influence of fiber alignment and diameter on the human pericyte growth. The presence of slightly elongated cells after 24 h of culture suggested that pericytes readily adhered to the PU scaffolds, although the fiber diameter and alignment did not affect cell coverage within the first 3 days of culture (30–40% for all analyzed materials). However, changes in cell morphology were observed after 3 days of culture, whereby pericytes grown on aligned fibers were more elongated and their mutual alignment was more uniform. Significant differences in cell growth and morphology between aligned and nonaligned materials were observed after 7 days of cell culture. Pericytes on aligned scaffolds were elongated, homogeneously oriented, and created dense layers, whereas nonaligned materials were not fully covered by the less elongated cells. Regarding fiber diameter, cell coverage was significantly higher on materials with a fiber diameter of 1000 nm (96–99%) in comparison to 500 nm (70–90%). The results demonstrated that fibrous PU scaffolds supported the pericyte growth. Moreover, pericyte proliferation was better on scaffolds with larger average fiber diameters, which is also characteristic of SMCs, as shown in our previous study.^26^

In this work, we examined the influence of fiber alignment on pericyte growth, and the results showed that the tested cells (pericytes) grow better on aligned fibers. The mechanism by which some cell types grow better on aligned fibers is not fully understood. Davidson et al. stated that mechanical intercellular communication between cells ensures stable cell–cell connections and proper tissue formation. Aligned fibers are one of the factors supporting mechanical intercellular communication. Aligned topography may promote contact guidance cues and enhance force transmission between cells, which enable cell directional extension and migration toward each other.^53^ Additionally, Fee et al. examined the influence of fiber alignment on genes expression. Performed analysis showed that the fibers alignment results in “upregulated gene expression in fibroblasts, especially the genes associated with actin production, actin polymerization and focal adhesion formation”.^54^ Jia et al. described that fiber alignment increases mechanical properties, morphological orientation and protein promotion of smooth muscle cells.^55^ Mural cells such as smooth muscle cells and pericytes are characterized by similar phenotypes; thus, the better growth of pericytes on aligned fibers observed in this work may arise from better mechanical intercellular communication and gene/protein expression.

## 5 Conclusions

This study aimed to evaluate the influence of the collector rotational speed on the physical and mechanical properties of PU scaffolds produced using the SBS method. The results showed that obtaining aligned PU fibers is limited by the fiber diameter, as homogeneously oriented fibers were achieved only for fiber diameters of ≥500 nm. Moreover, variations in fiber orientation and fiber diameter were more noticeable for materials with an average fiber diameter of 1000 nm in comparison to 500 nm, which suggests that a greater fiber diameter enables better control over the scaffold. The mechanical properties of the produced materials strongly depend on the direction of stretching, but the orientation of the fibers influences the mechanical strength only for materials with a fiber diameter of 1000 nm. The results further demonstrated that pericyte growth was improved on scaffolds with aligned fibers and the largest average fiber diameter (1000 nm) in the tested range.

In summary, by appropriately setting the SBS process parameters, scaffolds can be easily adapted to the cell requirements, which is of great importance in the production of complex 3D structures for guided tissue regeneration. Obtained results can be used for controlled design and production of scaffolds that act as a guide for the favorable regeneration of tissues. Materials with aligned fibers can be produced in tubular form as guiding scaffolds for vascular vessels regeneration.

## Supporting Information Available

Figure S1, fiber deviation angle distributions, *n* = 150; Figure S2, materials thickness, AVR ± SD, *n* = 90; Figure S3, representative SEM images of cross-sections of materials produced at collector rotation speeds of (A) 200 rpm, (B) 400 rpm, (C) 1 000 rpm, (D) 5000 rpm, (E) 10 000 rpm, (F) 15 000 rpm, (G) 20 000 rpm, and (H) 25 000 rpm at a magnification of 300×; Figure S4, fiber diameter distributions, *n* = 100 (PDF)
