# Supplementary material for: Characterization of Blow-Spun Polyurethane Scaffolds–Influence of Fiber Alignment and Fiber Diameter on Pericyte Growth
Source: ACS Biomater Sci Eng. 2024 Jun 10;10(7):4388–99. doi: 10.1021/acsbiomaterials.4c00051 (PMC11234331; doi:10.1021/acsbiomaterials.4c00051)
Supplement: Supplementary file 1 — ab4c00051_si_001.pdf [file ab4c00051_si_001.pdf]

Characterization of blow-spun polyurethane scaffolds – influence of fiber alignment and fiber diameter on pericyte growth

Iwona Łopianiak[1,2,], Aleksandra Kawecka[1], Mehtap Civelek[3], Michał Wojasiński[1], Iwona Cicha[3], Tomasz Ciach[1], Beata A. Butruk-Raszeja[1]*

[1] Laboratory of Biomedical Engineering, Faculty of Chemical and Process Engineering, Warsaw University of Technology, Waryńskiego 1, 00-645 Warsaw, Poland

[2] Doctoral School of Warsaw University of Technology, Plac Politechniki 1, 00-661 Warsaw, Poland

[3] Section of Experimental Oncology und Nanomedicine (SEON), Else Kröner-Fresenius-Stiftung-Professorship, ENT-Department, Universitätsklinikum, GluckstraBe 10a, 91054 Erlangen, Germany

*Corresponding author email adress: iwona.lopianiak.dokt@pw.edu.pl

AUTHOR INFORMATION

*Corresponding Author

Iwona Łopianiak – Laboratory of Biomedical Engineering, Faculty of Chemical and Process Engineering, Warsaw University of Technology, Waryńskiego 1, 00-645 Warsaw, Poland, iwona.lopianiak.dokt@pw.edu.pl

Authors

Aleksandra Kawecka – Laboratory of Biomedical Engineering, Faculty of Chemical and Process Engineering, Warsaw University of Technology, Waryńskiego 1, 00-645 Warsaw, Poland

Mehtap Civelek – Section of Experimental Oncology und Nanomedicine (SEON), Else Kröner-Fresenius-Stiftung-Professorship, ENT-Department, Universitätsklinikum Erlangen, Glueckstraβe 10a, D-91054 Erlangen, Germany

Michał Wojasiński – Laboratory of Biomedical Engineering, Faculty of Chemical and Process Engineering, Warsaw University of Technology, Waryńskiego 1, 00-645 Warsaw, Poland

Iwona Cicha – Section of Experimental Oncology und Nanomedicine (SEON), Else Kröner-Fresenius-Stiftung-Professorship, ENT-Department, Universitätsklinikum Erlangen, Glueckstraβe 10a, D-91054 Erlangen, Germany

Tomasz Ciach – Laboratory of Biomedical Engineering, Faculty of Chemical and Process Engineering, Warsaw University of Technology, Waryńskiego 1, 00-645 Warsaw, Poland

Beata A. Butruk-Raszeja – Laboratory of Biomedical Engineering, Faculty of Chemical and Process Engineering, Warsaw University of Technology, Waryńskiego 1, 00-645 Warsaw, Poland

SUPPLEMENTARY INFORMATION

Figures

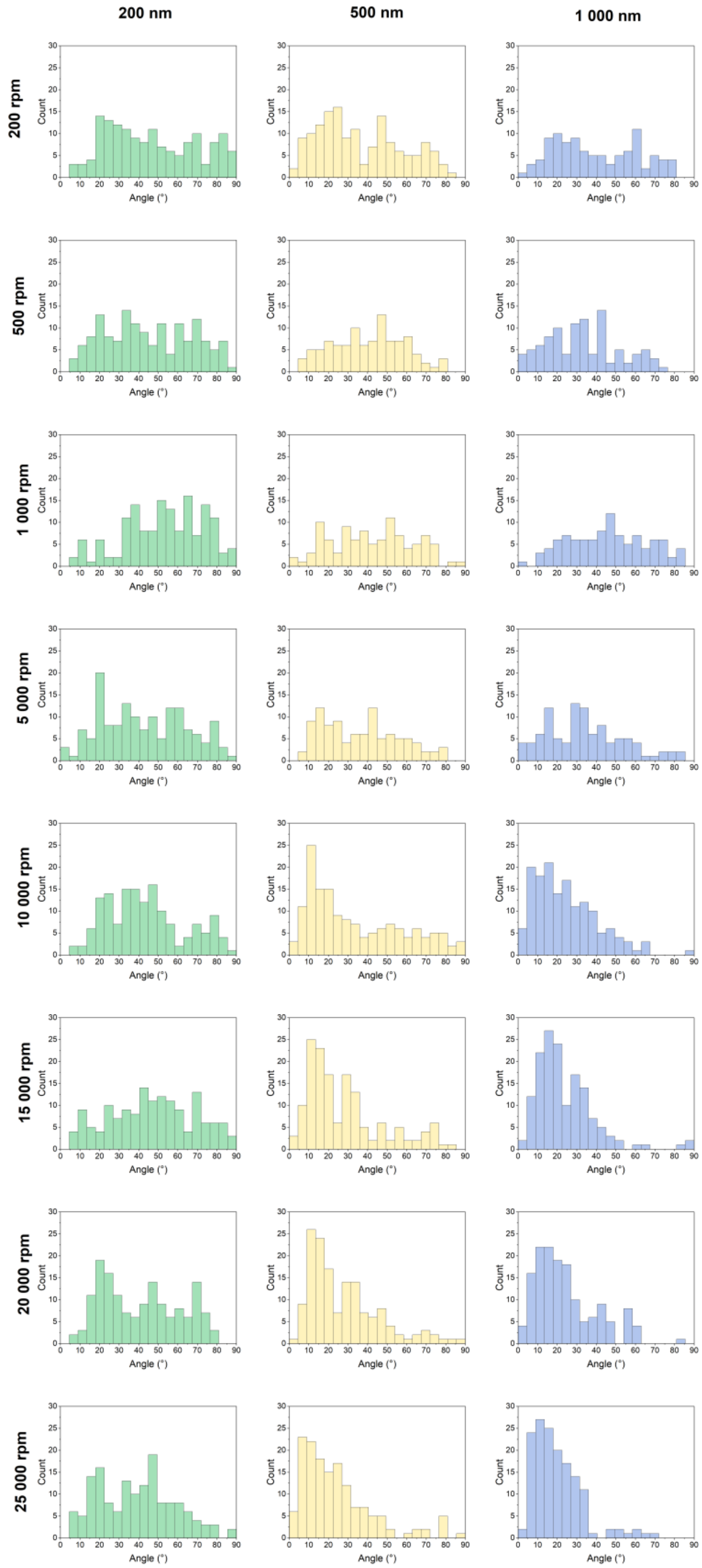

Figure S1 Fiber deviation angle distributions, n=150

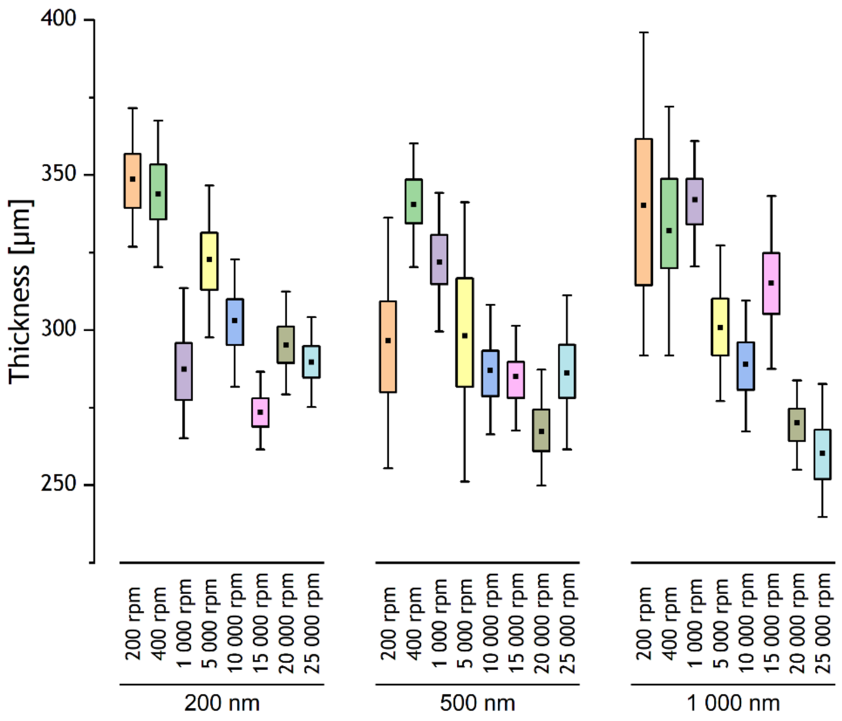

Figure S2 Materials thickness, AVR ± SD, n=90

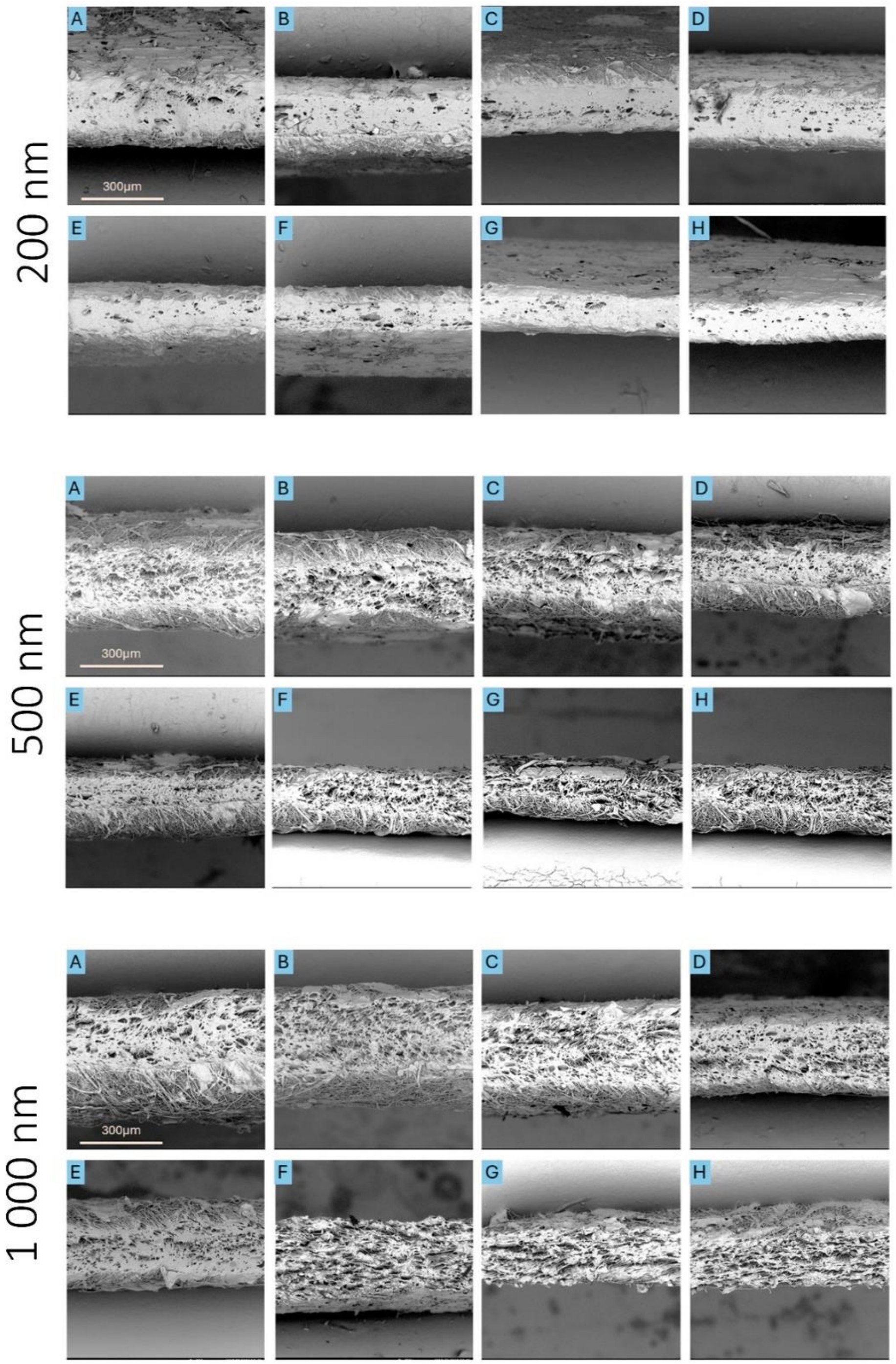

Figure S3 Representative SEM images of cross-sections of materials produced at collector rotation speeds of (A) 200 rpm, (B) 400 rpm, (C) 1 000 rpm, (D) 5 000 rpm, (E)10 000 rpm, (F) 15 000 rpm, (G) 20 000 rpm, and (H) 25 000 rpm at a magnification of x300.

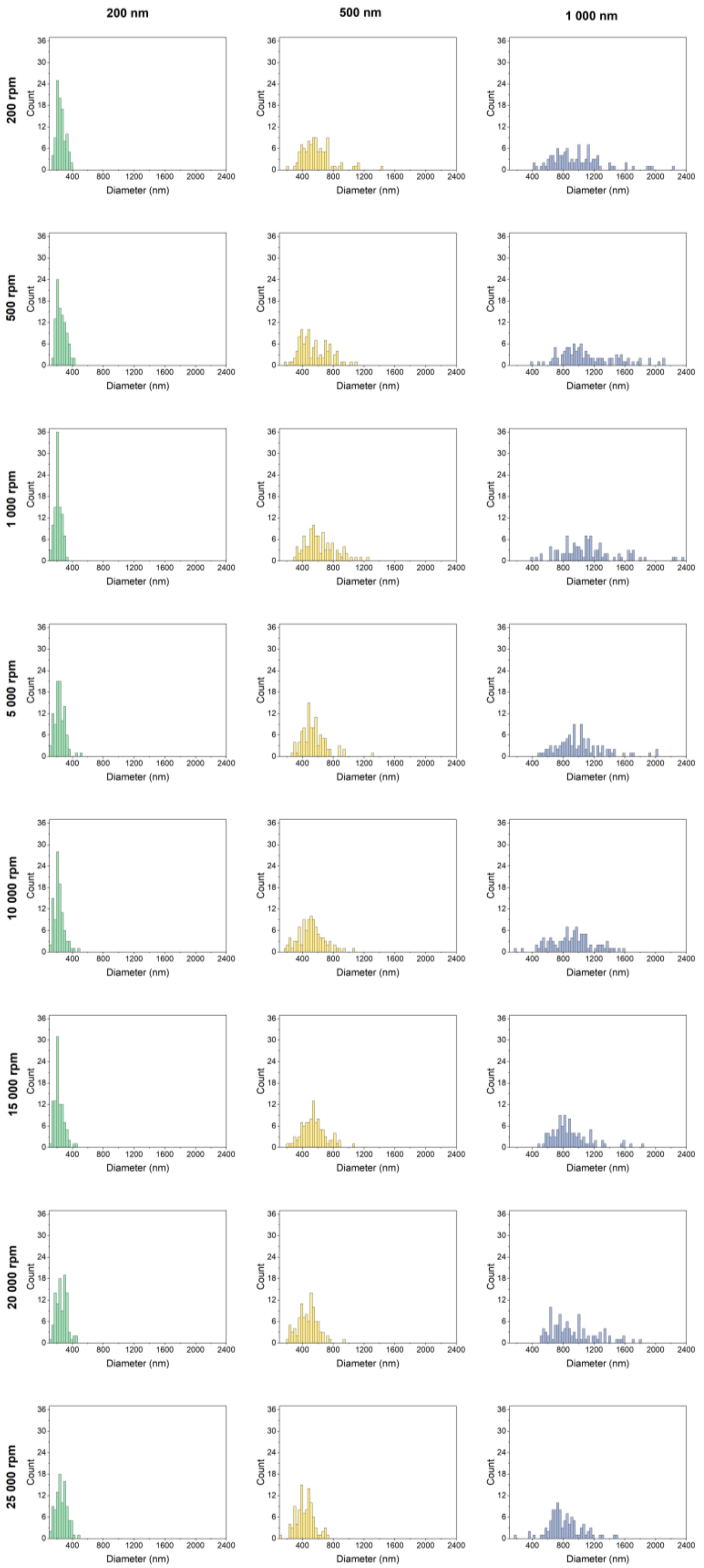

Figure S4 Fiber diameter distributions, n=100
